# Functional Outcomes Following Total Knee Arthroplasty Without Patellar Resurfacing: A Minimum Two-Year Follow-Up Retrospective Cohort Study

**DOI:** 10.7759/cureus.16036

**Published:** 2021-06-29

**Authors:** Daniel Li, Andrew S Bi, Sahej S Samra, Nehal S Samra, Decheng Wu, Yuangzheng Ma

**Affiliations:** 1 Orthopedics, The Ohio State University Wexner Medical Center, Columbus, USA; 2 Orthopaedic Surgery, New York University Langone Health, New York, USA; 3 Orthopaedic Surgery, Northwestern University Feinberg School of Medicine, Chicago, USA; 4 Medicine, Chinese Academy of Sciences, 301 PLA Military Hospital, Beijing, CHN; 5 Orthopaedic Surgery, Second Affiliated Hospital of Guangzhou Medical University, Guangzhou, CHN

**Keywords:** knee osteoarthritis, patellar resurfacing, post-operative outcomes

## Abstract

This paper seeks to address the effectiveness of total knee arthroplasty (TKA) when performed without patellar resurfacing. The objective of this article is to investigate the effect of total knee arthroplasty without patellofemoral resurfacing on postoperative outcome.

All patients with degenerative knee osteoarthritis (OA) that underwent TKA without patellar resurfacing were included in the study. The clinical data of 163 patients, including 98 females and 65 males with a mean age of 63 years (range 54-78 years) were retrospectively analyzed from April 2008 to April 2011. Intraoperative cartilage degeneration according to Outerbridge classification criteria was as follows: 22 cases of grade I, 38 cases of grade II, 64 cases of grade III, 39 cases of grade IV. There were no significant differences in gender, age, and side differences between the patients at all levels (P > 0.05). The duration of tourniquet use and related complications were recorded. Knee function was assessed using the American Knee Society Scoring System (KSS) and the patellar score (PS). Patient satisfaction and knee pain were assessed by the pain visual analog scale (VAS). The evaluation was conducted using routine X-ray film to observe the position of the prosthesis and the patella. Statistical analysis used included a comparison between groups by analysis of variance (ANOVA) using the Student-Newman-Keuls (SNK) test and comparison of grade data using the rank-sum test.

The average tourniquet time was 125 minutes, with a range of 90-150 minutes. All the incisions healed with primary intention without early complications. All patients were followed for two to five years with an average of 3.6 years. At six months and at the last follow-up, the KSS and PS scores were significantly higher than those before surgery (P < 0.05). There was no significant difference between the sixth month and the last follow-up (P > 0.05). There were significant differences in preoperative KSS and PS scores between patients with different grades of cartilage degeneration (P < 0.05), but there was no significant difference at the last follow-up (P > 0.05). At the last follow-up, seven patients had persistent anterior knee pain, five patients had mild pain, and two patients had moderate pain according to the VAS assessment criteria. Patient satisfaction evaluation was as follows: 90 patients were very satisfied, 66 patients were satisfied, five patients were uncertain, and two patients were unsatisfied. There were no significant differences in satisfaction and knee pain between patients with different grades of patellofemoral degeneration (P > 0.05).

In conclusion, at six months and at the last follow-up, outcome measures for patients were significantly higher than before surgery for TKA without the use of patellar resurfacing and the majority of patients were satisfied with the outcome of the procedure. TKA continues to be a successful procedure without the use of patellar resurfacing.

## Introduction

Total knee arthroplasty (TKA) is one of the most effective methods for the treatment of knee arthritis. However, there is currently no consensus on whether patellar resurfacing significantly affect postoperative outcomes [1]. A considerable percentage (6%-25%) of patients undergoing primary TKA with and without patellar resurfacing report postoperative anterior knee pain, and there are notable differences related to the surgical options between countries. For example, in North America, Denmark, and Australia, 90%, 80%, and 60% of surgeons resurface the patella, respectively, while only 2% perform the procedure in Sweden and Norway [2].

While some studies have shown that the incidence of anterior knee pain and reoperation rate in patients with patellar resurfacing is lower than in patients without patellar resurfacing [3], complications following patellar replacement such as periprosthetic fracture, avascular necrosis, aseptic loosening, and postoperative infection are higher [4-6]. Studies also demonstrate that the incidence of patellofemoral clunk in the resurfaced group was significantly greater than in the group without patellar resurfacing [7]. Advocates of leaving the patella alone argue that patellar resurfacing provides no advantages in functional outcomes, reoperation rate, or overall healthcare cost, and is instead linked to more complications [8]. Conversely, non-resurfacing appears to be associated with a higher likelihood of anterior knee pain, readmission, and additional interventions, but it is also related to a higher number of reoperations, which may be related to the surgeon's tendency to revise a non-resurfaced patella [5,9]. However, these past studies are not able to clarify the discrepancy in patellar resurfacing rates among surgeons in North America and Europe [10]. Most studies have suggested that the replacement of the patella during TKA should be based on the individual patient's condition and the severity of patellofemoral degeneration [11-12], but this recommendation continues to lack clear selection criteria and treatment guidelines.

The purpose of this study is to investigate postoperative outcomes of TKA when performed without patellofemoral arthroplasty. The authors hypothesize that the overall outcomes of procedures without patellofemoral arthroplasty will be satisfactory.

## Materials and methods

Patient selection criteria

We retrospectively analyzed 163 non-resurfaced patellas in TKAs performed at the Affiliated Tumor Hospital of Xinjiang Medical University from April 2008 to April 2011. Inclusion criteria were as follows: a diagnosis of degenerative osteoarthritis of the knee, surgeries were performed at the Center of Orthopedics at the 309th Hospital of PLA, Beijing, 100091 in China by surgeons in the same faculty practice group, unilateral primary TKAs using the Gemini MK II cemented prosthesis (Link, Germany), no patellar resurfacing, routine functional exercise was performed after surgery, and follow-up time was greater than or equal to two years. Exclusion criteria included: indications for TKA other than osteoarthritis, knee varus or valgus deformity greater than 15°, ipsilateral hip joint disease, low back pain, or psychiatric disorders, or non-compliance with the investigators. The study was approved by the Ethics Committee of the Affiliated Tumor Hospital of Xinjiang Medical University.

Surgical methods

Under general anesthesia, patients were positioned supine, with a tourniquet. A standard medial parapatellar approach was performed for exposure, and then all bony cuts, ligament balancing, implant insertion, and cement fixation were performed according to the Gemini MK II cemented prosthesis operative technique guide. At the end of cementing, the tourniquet was deflated and hemostasis was achieved with electrosurgery. Analgesia was injected around the joint using previously described “cocktails” following routine surgery [13]. These "cocktails" included 30 ml total of intraarticular injection consisting of 20 ml of 0.5% bupivacaine local anesthetic, 1 ml of morphine (10 ml), 0.3 ml of epinephrine, and 9.7 ml of normal saline (0.9% NaCl). A standard layered closure was performed, covered with a dressing and cotton pad, and pressure-bandaged.

Perioperative management

Prophylactic second-generation cephalosporin antibiotics were administered 30 minutes to one hour prior to anesthetic induction and continued for 24 hours following surgery. Appropriate postoperative deep venous thrombosis (DVT) prophylaxis of the lower extremities was given to all patients. Analgesia was maintained with celebrex for four weeks postoperatively. On postoperative day one, patients were encouraged to perform high-straightening quadriceps functional exercises, muscle isometric contraction exercises, and continuous passive motion (CPM) functional exercises.

Data collection

The tourniquet time during operation and the occurrence of related complications were recorded. Knee joint function was assessed using the American Knee Society Scoring System (KSS) [13] and the patellar score (PS) [14]. Patient satisfaction and knee pain were assessed by the pain visual analog scale (VAS). Satisfaction and pain were divided into four grades. Satisfaction was divided into very satisfied (> 8 points), satisfactory (6-8 points), uncertain (3-5 points), and unsatisfactory (0-2 points). The pain was divided into no pain (0-2 points), mild pain (3-5 points), moderate pain (6-8 points), and severe pain (> 8 points). Routine X-ray films were taken according to the X-ray film evaluation standard established by the American Knee Association [15]. The position of the patella was evaluated according to the patellofemoral joint physical examination protocol delineated by Lester et al. [16].

Statistical methods

All patient data were analyzed using SPSS Inc. (Version 16.0, Chicago, IL). The measurement data were expressed as mean ± standard deviation. The comparison between groups was analyzed by analysis of variance (ANOVA). The comparison between the two groups was performed using the Student-Newman-Keuls (SNK) test. The comparison of grade data was performed using the rank-sum test. The test level was α=0.05.

## Results

Patient information

A total of 163 patients met the selection criteria for inclusion in the study. This group contained 65 males and 98 females whose ages ranged from 54 to 78 years, with an average of 63 years. The body mass index (BMI) ranged from 19 kg/m2 to 30 kg/m2 with an average of 24 kg/m2. All patients suffered from degenerative osteoarthritis of one knee, including 54 cases of the left knee and 109 cases of the right knee. The course of disease ranged from two to eight years with an average of five years. All patients had pain around the knee joint and experienced limited mobility, and in all cases, more conservative treatment was found to be ineffective. Upon admission, the examination gave the following: knee joint range of motion (ROM) arcs ranged from 50 to 100°, with an average of 80°. According to the Outerbridge grading criteria [17], the intraoperative patellar cartilage degeneration was as follows: 22 cases of grade I, 38 cases of grade II, 64 cases of grade III, and 39 cases of grade IV. There were no significant differences in gender, age, and side differences between the patients at all levels (P < 0.05) (Table 1).

**Table 1:** Comparison of preoperative general data among patients with different grades of cartilage degeneration

Group	n	Gender (male : female)	Age (years)	Side (left : right)	Disease duration (years)
Grade I	22	9 : 13	64.2 ± 4.3	7 : 15	3.1 ± 2.4
Grade II	38	15 : 23	64.8 ± 4.2	12 : 26	3.6 ± 2.7
Grade III	64	26 : 38	66.7 ± 3.4	23 : 41	4.8 ± 3.1
Grade IV	39	15 : 24	67.1 ± 4.5	12 : 27	5.7 ± 1.3
Statistic		χ2 = 0.05, P = 0.87	F = 3.43, P = 0.24	χ2 = 2.23, P = 0.57	F = 9.87, P = 0.02

Functional assessment

All patients underwent primary TKAs. Tourniquets were used for 90 to 150 minutes with an average of 125 minutes. No early complications occurred. All patients were followed up for two to five years with an average of 3.6 years. At six months and at the last follow-up, the KSS and PS scores were significantly higher than those before surgery (P < 0.05). There was no significant difference between the follow-up at six months and the last follow-up (P > 0.05) (Table 2). There were significant differences in preoperative KSS and PS scores between patients with different grades of patellar cartilage degeneration (P < 0.05), but there was no significant difference at the last follow-up (P > 0.05) (Table 3). At the last follow-up, seven patients (4.3%) had persistent anterior knee pain. According to the VAS assessment criteria, five patients had mild pain and two patients had moderate pain. Among them, four patients had abnormal patellar position without special treatment. The other patients had no anterior knee pain. In the evaluation of patient satisfaction, 90 cases were very satisfied, 66 cases were satisfied, five cases were uncertain, and two cases were unsatisfied; overall satisfaction was 95.7%. There was no significant difference in the satisfaction of patients with different patellar cartilage degeneration grades and knee pain (P > 0.05) (Table 4).

**Table 2:** Comparison of American Knee Society Scoring System (KSS) and patellar scores (PS) between pre- and post-operation (n = 163, x̅ ± s) *Compared with preoperative value, P < 0.05; ^†^compared with the value at 6 months after operation, P < 0.05

Time	KSS	PS
Preoperative	84.7 ± 9.8^†^	11.5 ± 3.3^†^
Six months after operation	156.5 ± 8.7*	25.4 ± 5.3*
Last follow-up	168.6 ± 7.8*	26.5 ± 3.5*
Statistic	F = 6.45, P = 0.00	F = 8.92, P = 0.00

**Table 3:** Comparison of American Knee Society Scoring System (KSS) and patellar scores (PS) among patients with different grades of cartilage degeneration at pre- and post-operation (x̅ ± s)

	KSS			PS		
Group	n	Preoperative	Last follow-up	Statistic	Preoperative	Last follow-up	Statistic
Grade I	22	84.9 ± 7.6	164.7 ± 14.5	t = 3.21, P = 0.00	11.4 ± 4.3	26.2 ± 3.2	t = 8.74, P = 0.00
Grade II	38	82.3 ± 11.2	166.1 ± 11.3	t = 6.87, P = 0.00	10.5 ± 3.4	26.1 ± 3.1	t = 9.32, P = 0.00
Grade III	64	78.4 ± 9.2	168.7 ± 15.6	t = 5.97, P = 0.00	9.3 ± 3.5	27.1 ± 4.1	t = 11.56, P = 0.00
Grade IV	39	74.4 ± 6.4	167.3 ± 13.7	t = 11.17, P = 0.00	8.3 ± 4.7	26.3 ± 3.2	t = 13.78, P = 0.00
Statistic		F = 4.35, P = 0.02	F = 2.43, P = 0.42		F = 5.66, P = 0.02	F = 2.36, P = 0.84	

**Table 4:** Comparison of satisfaction and anterior knee pain among patients with different grades of cartilage degeneration at last follow-up

		Satisfaction				Anterior knee pain			
Group	n	Very satisfied	Satisfied	Not certain	Not satisfied	No	Mild	Moderate	Severe
Grade I	22	8	13	1	0	21	1	0	0
Grade II	38	19	17	1	1	36	1	1	0
Grade III	64	43	18	2	1	61	2	1	0
Grade IV	39	20	18	1	0	38	1	0	0
Statistic		χ^2 ^= 4.32, P = 0.09				χ^2 ^= 0.36, P = 0.10			

Radiographic evaluation

At postoperative day three, radiographic examination showed a femoral angle of 96.5° ± 2.4°, a tibial angle of 89.7° ± 0.7°, a posterior tilt angle of the tibial plateau of 87.3° ± 1.4°, and a femoral flexion angle of 1.1° ± 0.7°. All were within normal ranges. At the last follow-up, no osteolysis, periprosthetic fractures, or loosening of the prosthesis were observed in this group. There were no radiographic indications of osteonecrosis or fracture. In four cases, the patellar tracking trajectory was poor. One of the patellas was displaced 3 mm to the lateral side, and the third patella was tilted 5° to 7° laterally (average 5.7°). All four patients had different degrees of knee pain.

## Discussion

Overall, at six months and at the time of the last follow-up, outcomes for patients undergoing TKA without the use of patellar resurfacing were significantly improved than before surgery, and the majority of patients were satisfied with procedural outcomes. At six months and at the last follow-up, the KSS and PS scores were significantly higher than those before surgery, but there was no significant difference between the sixth month and the last follow-up. Moreover, there were significant differences in preoperative KSS and PS scores between patients with different grades of cartilage degeneration, but there were no significant differences at the last follow-up. At the last follow-up, the patient satisfaction rate was 95.7%, and the anterior knee pain rate was 4.3%. The KSS and PS scores were significantly higher than those before surgery, and the postoperative outcomes were similar among patients with varying degrees of patellar cartilage degeneration.

Historically, in the early stages of TKAs, patellar resurfacing was rarely performed by most surgeons. However, as the incidence of postoperative anterior knee pain began to increase in TKA patients, more and more surgeons began to perform patellofemoral arthroplasty under the assumption that it improved postoperative outcomes. Interestingly, due to this surge in patella replacement surgery, complications associated with patella replacement and associated revision surgery have increased [18]. To this end, some scholars believe that the patella should be selectively replaced according to the individual characteristics of the patient and the degree of degeneration of the cartilage and that only patients with Outerbridge grade IV patellar defects are indicated for patella replacement during TKA [12,19]. Furthermore, Longo et al. demonstrated in a detailed systematic review and meta-analysis that TKAs performed with patellar resurfacing have shown better outcomes than non-resurfaced TKAs, including significantly lower secondary operation and revision rates for the former group [20].

Yet, other studies have reached the opposite conclusion. Whiteside et al. found that the incidence of postoperative anterior knee pain was related to prosthesis design in patients with TKA retention, but there was no significant correlation with the degree of patellar cartilage degeneration [21]. Additionally, patellar resurfaced TKAs were associated with both greater anterior knee pain and a higher rate of revision in investigations by Pakos et al. and Calvisi et al. [5,22]. A systematic review conducted by Li et al. provided the conclusion that patellar resurfacing can reduce the risk of reoperation compared to TKA without resurfacing, but there is no evidence to suggest an improvement in postoperative knee function or patient satisfaction. Randomized controlled trials have been completed, but no conclusive evidence has been gathered concerning which option is superior [23-27].

There are several limitations to our study. Prior studies have shown that poor trajectory of the patella and the presence of painful afferent fibers in the soft tissue around the patella can also be responsible for knee pain, highlighting a potential confounding factor in this investigation [28]. To mitigate this possibility, electrosurgery was used around the patella to destroy the peripheral nerves and block neurogenic pain pathways. Still, of the seven patients with postoperative knee pain, four were due to poor patellar tracking, indicating a limitation of this study. Other shortcomings include the retrospective nature of this study and a lack of a control group to compare outcomes directly against TKAs performed with patellar resurfacing. A final area of weakness in the study design includes the fact that multiple surgeons conducted the surgeries, leading to varying operative techniques and post-operative rehabilitation protocols. In the future, as with many questions in orthopaedic surgery, high-quality, randomized controlled studies should be performed to best compare TKAs with versus without patellar resurfacing, and long-term efficacy needs to be followed over a longer postoperative time period.

## Conclusions

The main finding of this study is that performing TKA without patellar resurfacing does not prohibit significant increases in postoperative functional outcome scores. These results indicate that satisfactory clinical outcomes can be achieved in patients with degenerative knee osteoarthritis but intact patellofemoral compartments when performing a TKA.
